# The Value of Tumor Infiltrating Lymphocytes (TILs) for Predicting Response to Neoadjuvant Chemotherapy in Breast Cancer: A Systematic Review and Meta-Analysis

**DOI:** 10.1371/journal.pone.0115103

**Published:** 2014-12-12

**Authors:** Yan Mao, Qing Qu, Yuzi Zhang, Junjun Liu, Xiaosong Chen, Kunwei Shen

**Affiliations:** 1 Comprehensive Breast Health Center, Ruijin Hospital, Shanghai Jiao Tong University School of Medicine, Shanghai, China; 2 Department of Oncology, Ruijin Hospital, Shanghai Jiao Tong University School of Medicine, Shanghai, China; Harvard Medical School, United States of America

## Abstract

**Background:**

We carried out a systematic review and meta-analysis to evaluate the predictive roles of tumor infiltrating lymphocytes (TILs) in response to neoadjuvant chemotherapy (NAC) in breast cancer.

**Method:**

A PubMed and Web of Science literature search was designed. Random or fixed effect models were adopted to estimate the summary odds ratio (OR). Heterogeneity and sensitivity analyses were performed to explore heterogeneity among studies and to assess the effects of study quality. Publication bias was evaluated using a funnel plot, Egger's test and Begg's test. We included studies where the predictive significance of TILs, and/or TILs subset on the pathologic complete response (pCR) were determined in NAC of breast cancer.

**Results:**

A total of 13 published studies (including 3251 patients) were eligible. In pooled analysis, the detection of higher TILs numbers in pre-treatment biopsy was correlated with better pCR to NAC (OR = 3.93, 95% CI, 3.26–4.73). Moreover, TILs predicted higher pCR rates in triple negative (OR = 2.49, 95% CI: 1.61–3.83), HER2 positive (OR = 5.05, 95% CI: 2.86–8.92) breast cancer, but not in estrogen receptor (ER) positive (OR = 6.21, 95%CI: 0.86–45.15) patients. In multivariate analysis, TILs were still an independent marker for high pCR rate (OR = 1.41, 95% CI: 1.19–1.66). For TILs subset, higher levels of CD8+ and FOXP3+ T-lymphocytes in pre-treatment biopsy respectively predicted better pathological response to NAC (OR = 6.44, 95% CI: 2.52–16.46; OR = 2.94, 95% CI: 1.05–8.26). Only FOXP3+ lymphocytes in post-NAC breast tissue were a predictive marker for low pCR rate in univariate (OR = 0.41, 95% CI: 0.21–0.80) and multivariate (OR = 0.36, 95% CI: 0.13–0.95) analysis.

**Conclusion:**

Higher TILs levels in pre-treatment biopsy indicated higher pCR rates for NAC. TILs subset played different roles in predicting response to NAC.

## Introduction

Breast cancer is one of the most common malignancies among women all over the world. In the USA, approximately 230,000 new cases of invasive breast cancer are expected to be diagnosed in 2014 [1]. However, due to both early diagnosis and improved systemic therapy, the mortality rates for this kind of tumor have decreased in recent decades. Early stage breast cancer may be cured with the future development of therapeutic approaches that are based on appropriate biomarkers. The immune system has been a promising new target for breast cancer diagnosis. Indeed, a large body of evidence has shown the existence of immune defects in breast cancer patients, and various studies have observed the heavy infiltration of tumors by immune cells [2], [3]. These immune cells are primarily tumor-infiltrating lymphocytes (TILs) that are associated with good prognosis in various cancers, such as epithelial ovarian carcinoma [4], [5], endometrial cancer [6]–[10], and also breast cancer [11]–[14]. These cells demonstrate that the host immune response plays an important role in tumor progression.

Systemic neoadjuvant therapy is the treatment of choice for patients with locally advanced breast cancer and is increasingly used to treat patients with operable breast cancer who are not candidates for breast-conserving surgery or who have proven lymph node metastases[15], [16]. Although both chemotherapy and endocrine therapy have been administered in the neoadjuvant setting, cytotoxic chemotherapy is more commonly used because of a more extensive and rapid response. Anti-HER2 therapy, such as trastuzumab, has also been administered in the neoadjuvant setting in combination with chemotherapy in HER2 positive patients [17]. More importantly, patients showing a pathologic complete response (pCR) to neoadjuvant chemotherapy (NAC) may experience prolonged disease-free survival, especially in triple negative breast cancer patients [18]–[20]. Therefore, identifying effective biomarkers useful for predicting the pCR rate is a high priority.

Previous studies have shown that the changes in Ki67 before and after neoadjuvant chemotherapy may indicate a higher pCR rate and good prognosis for breast cancer [21]–[23]. Also, there are some studies showing that infiltration of lymphocytes in tumor may have predictive values for NAC response [24]–[34] and indicate good survival in adjuvant setting [35], [36], but there are no confirmed results of their roles in predicting pCR rate in a neoadjuvant chemotherapy setting. Therefore, we performed a systematic review and meta-analysis, aiming to establish pooled estimates for pCR rate based on the presence of TILs in breast cancer and different subtypes. Since many studies identified TILs by CD3, CD4, CD8 and FOXP3, we also analyzed the predictive value of TILs subset in response to NAC.

## Methods

### Search strategy

Original articles studying the predictive value of TILs in the neoadjuvant setting of breast cancer were sought in the PubMed and Web of Science databases using the following key words: ‘breast cancer’, ‘lymphocytes, tumor-infiltrating’, ‘CD3-positive T-Lymphocytes’, ‘CD4-positive T-Lymphocytes’, ‘CD8-positive T-Lymphocytes’, ‘FOXP3-positive T-lymphocytes’ ‘neoadjuvant’ and ‘pathologic complete response’. Additionally, possible additional articles were searched in reference lists of selected papers and related articles as suggested by PubMed. Review articles were also scanned for additional eligible studies.

### Inclusion and exclusion criteria

Original and review articles published before May 2014 were extracted. The search results were then screened according to the following inclusion criteria: (1) published as original articles; (2) evaluated human subjects;(3) investigated the predictive value of TILs, CD3+, CD4+, CD8+, and FOXP3+ lymphocytes, including ratios between these subsets in neoadjuvant chemotherapy settings**;** (4) reported the relationship between TILs and pCR rates; (5) contained the minimum information necessary to estimate the effects (i.e., odds ratio) and a corresponding measure of uncertainty (i.e., confidence interval, P-values, standard errors or variance); (6) written in English. As an additional criterion, when a single population was reported in multiple reports, only the report with the most complete data was included to avoid duplication.

Articles were excluded from the analysis if they met the following criteria: (1) non-original reports; (2) in vitro and animal study; (3) absence of key information such as, odds ratio (OR), 95%CI and P value; (4) immunological clinical trials were rejected, because active immunotherapy aims to modify the presence or the composition of T-lymphocyte subsets.

### Data extraction and quality assessment

The selected articles were assessed independently by two reviewers (Y.M. and Q.Q.). The following data were collected from each of the included studies and key elements pertaining to the study design, sample size, country, pCR definition, T lymphocyte subsets, T lymphocyte counting sites, treatment regimen, use of multivariate or univariate logistic model analysis, OR estimates (with the corresponding 95% CIs) for the high density over the low TILs density or each T-lymphocytes subset at certain locations within tumors (intratumoral, stromal and both sites) and the staining cutoff point were obtained (Table 1, S2 Table, S3 Table). Discrepancies were resolved by discussion with a third reviewer (Y.Z.Z.) or by contacting content experts if necessary until the two reviewers reached consensus.

**Table 1:** Baseline Characteristics of Included Studies.

Authors and published years	Data collection	Pts No	Country of origin	Ethnicity	pCR definition	Cut-off value	Assay	Marker	Time	Location	Neoadjuvant regimen
Ladoire et al. 2008 [27]	Retrospective	56	France	Europe	ypT0	Scores>0	IHC	CD3, CD8, FOXP3	Pre-,post-NAC	IS	CEF,CEX, H,,DC
Aruga et al. 2009 [24]	Retrospective	87	Japan	Asia	Japanese criteria	Median	IHC	FOXP3	Pre-,post-NAC	IS	CEF,EC,EC-T,CEF-T
Denkert et al. 2010a [25]	Prospective	218	Germany	Europe	ypT0/TisypN0	10%,LPBC,10% INC	H&E	TILs	Pre-NAC	IS,SS	EC-T
Denkert et al. 2010b [25]	Prospective	840	Germany	Europe	ypT0/TisypN0	10%,LPBC,10% INC	H&E, IHC	TILs, CD3	Pre-NAC	IS,SS	TAC, NX
West et al. 2011 [33]	Prospective	113	Europe	Europe	Not clear	upper quartile	Gene-signature	TILs	Pre-NAC	BS	CEF,TET
Oda et al. 2012 [30]	Retrospective	180	Japan	Asia	ypT0 ypN0	median	IHC	CD8, FOXP3	Pre-,post-NAC	IS	CEF-T
Ono et al. 2012 [31]	Retrospective	180	Japan	Asia	ypT0	score>2	H&E	TILs	Pre-NAC	BS	AC,ACT,CEF,AT, CEF-T
Yamaguchi et al. 2012 [34]	Retrospective	68	Japan	Asia	ypT0	Score>1	H&E	TILs	Pre-NAC	BS	CEF-T
Liu et al. 2012 [28]	Retrospective	132	China	Asia	ypT0	median	IHC	FOXP3	Pre-,post-NAC	SS	CEF,CEX
Seo et al. 2013 [32]	Retrospective	153	Korea	Asia	ypT0 ypN0	median	IHC	CD4,CD8, FOXP3	Pre-NAC	IS	AC,AC-T,AD
Lee et al. 2013 [42]	Retrospective	175	Korea	Asia	ypT0 ypN0	10%,10% INC	IHC	CD3,CD8,FOXP3	Pre-NAC	SS	AT,AC-T,H
Loi et al. 2013 [43]	Retrospective	156	Germany	Europe	ypT0, ypTis	10% INC	H&E	TILs	Pre- NAC	SS	EC-TX, H,EC-T
Denkert et al. 2013 [41]	Prospective	580	Germany	Europe	ypT0ypN0	LPBC,10% INC,	H&E	TILs	Pre-NAC	IS,SS	PM+/−Cb,Bev,H,L
Issa-Nummer et al. 2013 [26]	Prospective	313	Germany,Switzerland	Europe	ypT0ypN0	10% INC	H&E	TILs	Pre- NAC	IS,SS	EC-T,Bev,EVE, L

Abbreviations: pts, patients; IHC, immunohistochemistry; HE, staining Hematoxylin-eosin staining; FOXP3, regulatory T-lymphocytes expressing forkhead box P 3 protein; NAC, neoadjuvant chemotherapy; CEF, cyclophosphamide/epirubicin/fluorouracil; CEX, epirubicin/cyclophosphamide/capecitabine; DC, docetaxel/carboplatin; EC, epirubicin/cyclophosphamide; EC-T, epirubicin/cyclophosphamide-taxane; CEF-T, cyclophosphamide/epirubicin/fluorouracil-taxane;T AC, docetaxel/doxorubicin/cyclophosphamide; NX, vinorelbine/capecitabine; TET, docetaxel-docetaxel/epirubicin; AC, doxorubicin/cyclophosphamide; AC-T, doxorubicin/cyclophosphamide-taxane; AD, doxorubicin/docetaxel; AT, doxorubicin/taxane; EC-TX, epirubicin/cyclophosphamide-docetaxel/capecitabine; EC-T-X, epirubicin/cyclophosphamide-docetaxel-capecitabine; PM, paclitaxle/non-pegylated liposomal doxorubicin; PMCb, paclitaxle/non-pegylated liposomal doxorubicin/carboplatin; H, trastuzumab; Bev, bevacizumab; L, lapatinib; EVE, everolimus; IS =  Intratumoral sites; SS =  stromal sites;BS, both sites;10% INC, 10 incr**em**ent

a, GeparDuo.

**b**, GeparTrio.

The quality of each study was assessed using an established form that was first developed and applied by McShane et al. [37] and Hayes et al. [38]. The following seven domains were assessed and scored on a scale from 0 to 8: inclusion and exclusion criteria, study design (prospective or retrospective), patient and tumor characteristics, description of the method or assay, study endpoints, follow-up time with patients and the number of patients that dropped out during the follow-up period. Studies achieving five or more scores were considered to be high quality.

### Statistical analysis

We evaluated the overall ORs and 95% CIs of eligible data for the predictive value of TILs in pCR to NAC in breast cancer. The OR extracted from each study provided an estimate of the ratio of pCR rate for high-density vs. low-density of TILs and/or TILs subset. We then performed subgroup analyses according to the location of lymphocytes infiltration, breast cancer subtypes and TILs subset (CD3+, CD4+, CD8+, or FOXP3+). Pooled ORs were obtained using the chi-square based Q test for heterogeneity assuming two models. Presence of heterogeneity (p<0.05 or I^2^>50%) merited use of the random effects model, the fixed-effects model was used in its absence (p>0.05 or I^2^<50%). Heterogeneity between studies was evaluated using sensitivity analysis. Publication bias was evaluated using the funnel plot with the Egger's [39] and Begg's [40] tests. All statistical analyses were performed using STATA version 11.0 (Stata Corporation, College Station, TX, USA).

## Results

### Search results

**Figure 1:**
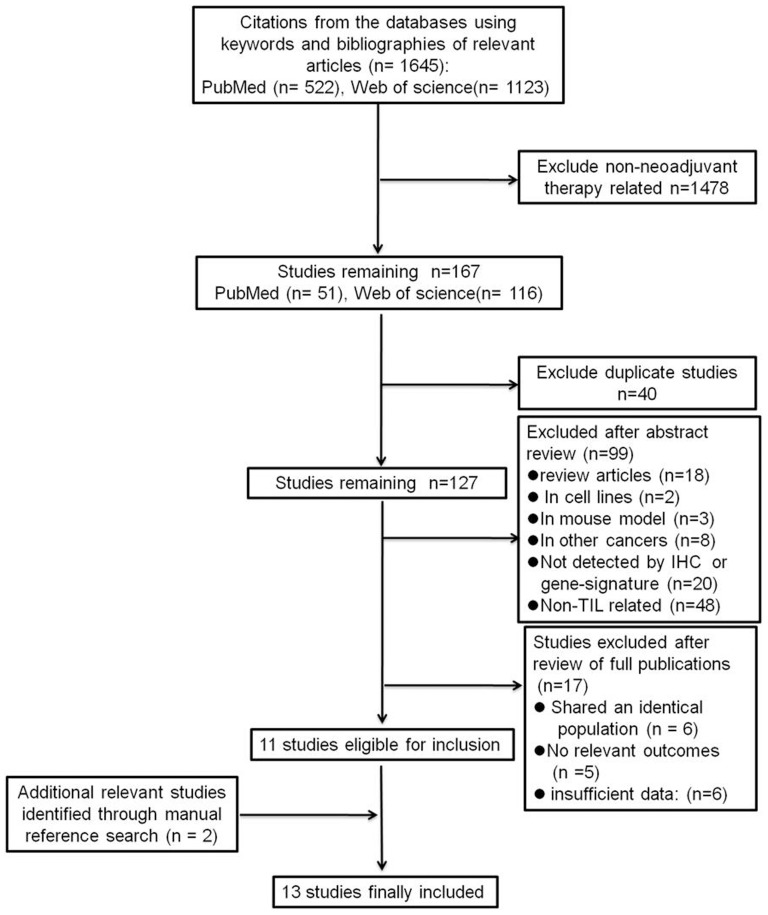
Flow chart for the selection process of eligible publications.

Fig. 1 presents the selection process for eligible studies. Briefly, a total of 1647 studies were identified for initial evaluation, after a series of exclusions, the final number of studies included in the meta-analysis was 13 and involving 3251 patients. The agreement between the two authors was 98% for study selection and 93% for trial quality assessment**s**.

### Study characteristics

The characteristics of all included studies are summarized in Table 1. In the thirteen studies, generalized TILs were reported in eight studies [25], [26], [31], [33], [34], [41]–[43], and TILs subset were reported in seven studies [24], [25], [27], [28], [30], [32], [42] including two studies evaluated both TILs and subsets [25], [42]. Six [25]–[27], [33], [41], [43] and seven [24], [28], [30]–[32], [34], [42] studies were performed in Europe and Asia, respectively. The total sample size from all studies were 3251, ranging from 56 to 840 patients, with a mean of 232 patients in each study. Ten studies enrolled more than 100 patients. All studies were published between 2008 and 2014. The eligible studies tested TILs before NAC (n = 9) or both before and after NAC (n = 4). The detection method for TILs was IHC (n = 6), H&E staining (n = 6) or gene-signature (n = 1). The majority of neoadjuvant chemotherapy regimen contains anthracycline and taxane. In HER2 positive patients, trastuzumab or lapatinib were typically used. The most frequently cutoff values used were 10% increment (n = 5), median (n = 4) and values calculated by semi-quantitative methods, such as the staining score (n = 3) or the upper quartile (n = 1). The most common definition of pCR in all studies was the absence of residual invasive tumor cells in breast and lymph nodes (n = 7), whereas the remaining 6 studies used the definition of no invasive cancer in breast (n = 2), no malignant cells in breast (n = 2), no malignant cells in both breast and axillary specimens (n = 1), or the Japanese pathological response criteria (n = 1). The assessment of quality for the individual study was presented in S1 Table. Seven studies had high quality with score more than 4, while six studies had low quality with score equals to or less than 4.

### Pooled analysis of TILs

**Figure 2:**
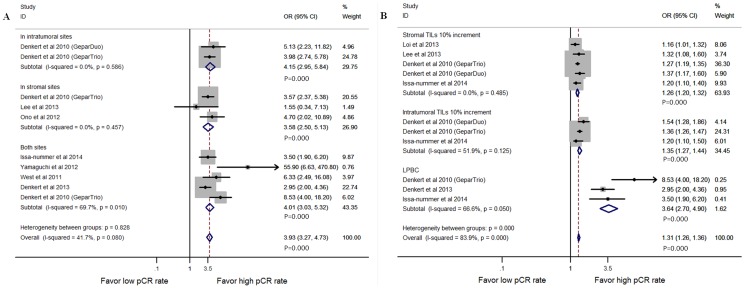
Forest plots from the fixed-effect meta-analysis of the efficacy of TILs on the NAC response stratified by infiltration locations (A) and different cutoff values (B). The width of horizontal line represents 95% CI of the individual studies, and the grey boxes represent the weight of each study. The diamond represents the overall summary estimate. The unbroken vertical line was set at the null value (HR = 1.0). Abbreviations: LPBC, lymphocyte-predominant breast cancer; TILs, tumor infiltrating lymphocytes.

Eight studies were pooled for analysis of the density of TILs in stromal, in intra-tumoral or both sites for pre-NAC biopsy in univariate way. These results indicated that TILs predicted a higher pCR rate for NAC (OR = 3.93, 95% CI, 3.26–4.73, P = 0.000), whether they were detected in intra-tumoral (OR = 4.15, 95% CI, 2.95–5.84, P = 0.000), stromal (OR = 3.58, 95% CI, 2.50–5.13, P = 0.000), or in both sites (OR = 4.01, 95% CI, 3.03–5.32, P = 0.000) (Fig. 2A). There were different cutoff values for TILs in these studies as we mentioned previously. Lymphocyte-predominant breast cancer (LPBC), defined as having more than 50 or 60% lymphocyte infiltration of the tumor bed or stroma, provided a very different cutoff relative to most studies. Therefore, we also analyzed the pooled OR in the three studies that tested the relationship between LPBC and the pCR rate in NAC [25], [26], [41]. The results showed that LPBC patients had higher pCR rates compare to non-LPBC patients, with an OR equals to 3.64 (95% CI, 2.70–4.90, P = 0.000; Fig. 2B). Another parameters evaluated the density of TILs was per 10% increase in the number of lymphocytes infiltrating in either intratumoral or stromal locations. They indicated a better pathological response to NAC with an OR equal to 1.35 (95% CI, 1.27–1.44, P = 0.000) and 1.26 (95% CI, 1.20–1.32, P = 0.000), respectively (Fig. 2B).

**Figure 3:**
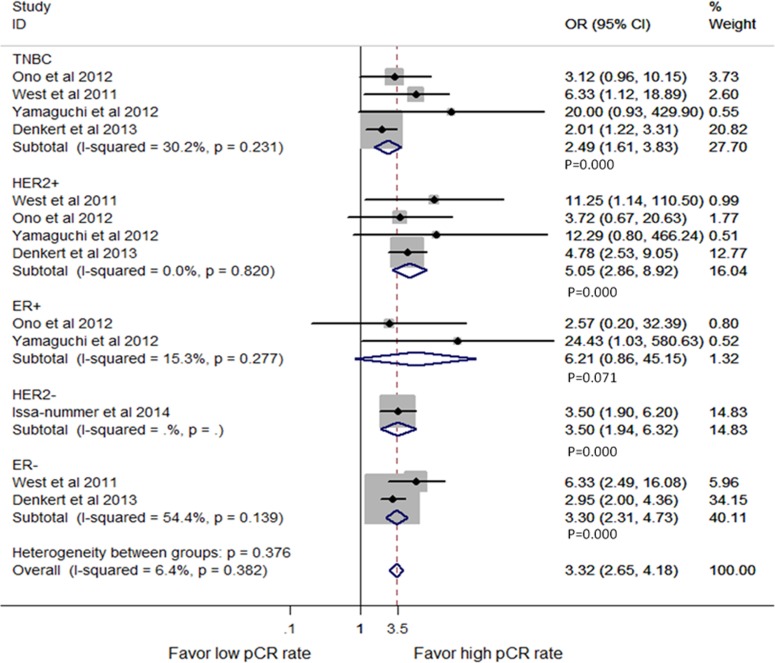
Forest plots from the fixed-effect meta-analysis of the efficacy of TILs on the NAC response stratified by different subtypes. The width of horizontal line represents 95% CI of the individual studies, and the grey boxes represent the weight of each study. The diamond represents the overall summary estimate. The unbroken vertical line was set at the null value (HR = 1.0). Abbreviations: TNBC, triple negative breast cancer; HER2, human epithelial growth factor receptor 2; ER, estrogen receptor.

Since breast cancer has been divided into four main molecular subtypes in clinical practice, we analyzed the predictive value of TILs in different subtypes. The analysis indicated that TILs tested before NAC had predictive values in ER negative (OR = 3.30, 95% CI, 2.31–4.73, P = 0.000), triple negative (OR = 2.49, 95% CI, 1.61–3.83, P = 0.000), HER2 positive (OR = 5.05, 95% CI, 2.86–8.92, P = 0.000) and HER2 negative patients (OR = 3.50, 95% CI, 1.90–6.20, P = 0.000) breast cancer patients, but not in ER positive patients (OR = 6.21, 95% CI, 0.86–45.15, P = 0.071; Fig. 3). However, limited studies analyzed the relationship between TILs and the pCR rate in these subtypes. Therefore, more prospective studies are needed.

**Figure 4:**
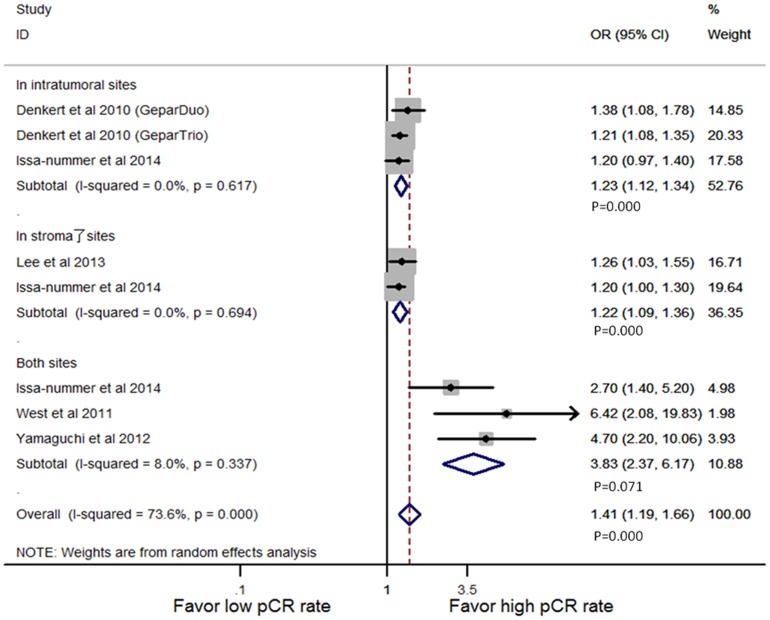
Forest plots from the random-effect meta-analysis of the efficacy of TILs on the NAC response stratified by infiltration locations in multivariate way. The width of horizontal line represents 95% CI of the individual studies, and the grey boxes represent the weight of each study. The diamond represents the overall summary estimate. The unbroken vertical line was set at the null value (HR = 1.0).

Considering age, tumor size, tumor type, grade, hormonal receptor status, lymph node status or therapeutic regimens, five studies [25], [26], [33], [34], [42] were pooled for analysis of the density of TILs by multivariate analysis. The cutoff values were 10% increment (in intra-tumoral and stromal sites) and LPBC (in both sites). The results indicated that TILs infiltration was an independent predictive marker for higher pCR rate (OR = 1.41, 95% CI, 1.19–1.66, P = 0.000), whether they were detected in intratumoral (OR = 1.23, 95% CI, 1.12-1.34, P = 0.000), stromal (OR = 1.22, 95% CI, 1.09–1.36, P = 0.000), or both sites (OR = 1.41, 95% CI, 1.19–1.66, P = 0.000; Fig. 4).

### Pooled analysis of TILs subset

**Figure 5:**
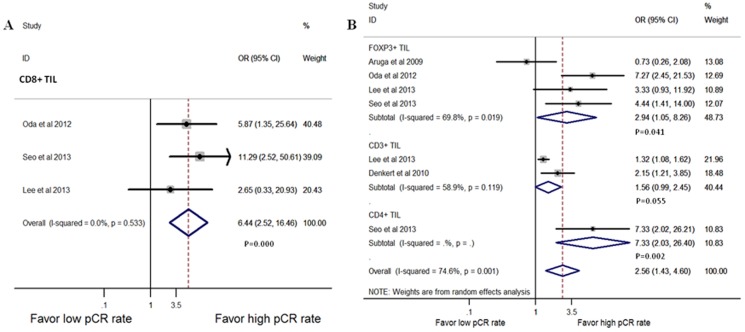
Forest plots from the fixed- or random-effect meta-analysis of the efficacy of TILs subset on NAC response in pre-treatment biopsy (A, B). The width of horizontal line represents 95% CI of the individual studies, and the grey boxes represent the weight of each study. The diamond represents the overall summary estimate. The unbroken vertical line was set at the null value (HR = 1.0).

**Figure 6:**
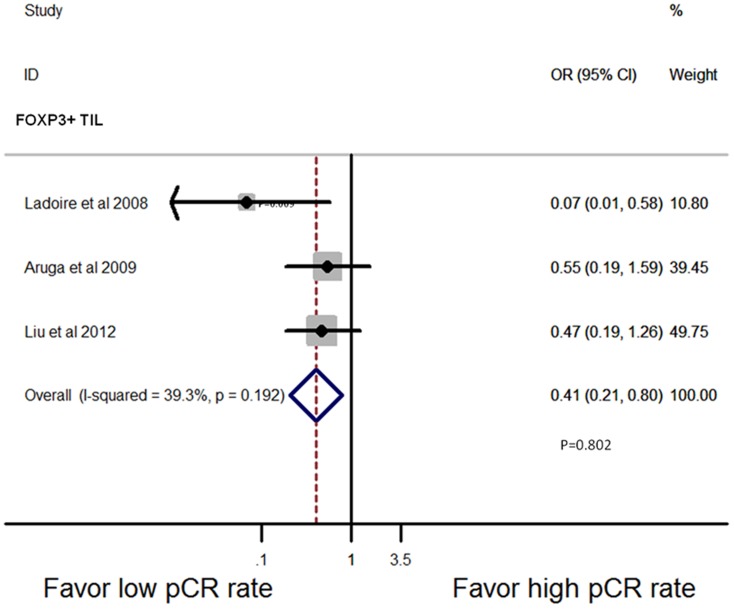
Forest plots from the fixed-effect meta-analysis of the efficacy of TILs subset on NAC response in post-treatment breast tissue. The width of horizontal line represents 95% CI of the individual studies, and the grey boxes represent the weight of each study. The diamond represents the overall summary estimate. The unbroken vertical line was set at the null value (HR = 1.0).

The most commonly tested markers for TILs subset tested in breast cancer are CD3+, CD4+, CD8+ and FOXP3+. Seven studies [24], [25], [27], [28], [30], [32], [42] were pooled for analysis of the association between T lymphocytes subsets and the pCR rate in univariate way. Five studies reported which subset were present in pre-treatment biopsy, and the pooled analysis showed that higher level of CD8+ (OR = 6.44, 95% CI, 2.52–16.46, P = 0.000; Fig. 5A) and FOXP3+ (OR = 2.94, 95% CI, 1.05–8.26, P = 0.041; Fig. 5B) lymphocytes indicated a better pathologic response to NAC, while the presence of CD3+ lymphocytes had no predictive values (OR = 1.56, 95% CI, 0.99–2.45, P = 0.055; Fig. 5B). Although CD4+ infiltrating lymphocytes also indicated a good response to NAC in pre-treatment biopsy (OR = 7.33, 95% CI, 2.03–26.40, P = 0.002; Fig. 5B), there is only one study tested this relationship. More prospective studies are needed to address this issue. Three studies [24], [27], [28] reported the presence of TILs subset in post-treatment breast tissue and indicated that higher levels of FOXP3+ lymphocytes infiltration after treatment indicated lower response to NAC (OR = 0.41, 95% CI, 0.21–0.80, P = 0.009; Fig. 6). Since there was not enough data available concerning the correlation of TILs subset in different locations and subtypes with NAC response, we were unable to perform further analysis.

**Figure 7:**
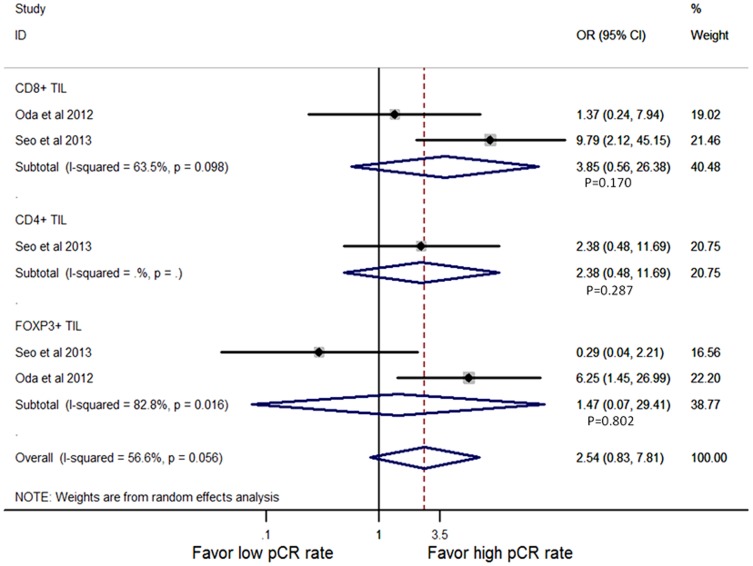
Forest plots from the random-effect meta-analysis of the efficacy of TIL subset on NAC response in pre-treatment biopsy in multivariate way. The width of horizontal line represents 95% CI of the individual studies, and the grey boxes represent the weight of each study. The diamond represents the overall summary estimate. The unbroken vertical line was set at the null value (HR = 1.0).

Considering age, HR status, HER2 status or Ki67 level, two studies [30], [32] were pooled for analysis of the density of TILs subset by multivariate analysis. These results indicated that TILs subset infiltration in pre-treatment biopsy was not an independent predictive marker for higher pCR rate (CD8+: OR = 3.85, 95% CI, 0.56–26.38, P = 0.170; CD4+: OR = 2.38, 95% CI, 0.48–11.69, P = 0.287; FOXP3+: OR = 1.47, 95% CI, 0.07–29.41, P = 0.802) (Fig. 7). Surprisingly, FOXP3+ lymphocytes infiltrated in breast tissue after treatment was an independent marker for pCR in one study (OR = 0.36, 95% CI, 0.13–0.95, P = 0.038) [28].

### Sensitivity analysis and publication bias

Sensitivity analysis for the predictive roles of TILs and TILs subset (CD8+, FOXP3+) in different study designs (prospective vs. retrospective), countries (European vs. Asia), sample sizes (≤100 vs.>100), study quality (score≤4 vs.>4), cutoff criteria (median vs. others) and locations (intratumoral, stromal or both sites) were shown in Table 2. The patterns of differences were similar to those of the original analysis. The heterogeneity among the studies was significantly reduced in studies from Europe, studies with prospective design, large sample size, high quality, 10% as a cutoff, intratumoral and stromal sites. For TILs subset, all studies included were retrospective. For CD8+ lymphocytes, all included studies had low quality (score  = 4). However, CD8+ cells in stromal sites or with 10% infiltration as a cutoff had no predictive values. All patients evaluated CD8 staining were from Asia, and there was no difference in studies from Japan or Korea. For FOXP3+ lymphocytes in pre-NAC breast biopsy, studies in Japan, with small sample size, median as a cutoff or in stromal, intratumoral sites did not exhibit significant differences in the predictive roles of FOXP3+ lymphocytes for pCR rate. For FOXP3+ lymphocytes in post-NAC breast tissue, studies in Asia, with a large sample size, or high quality did not exhibit significant differences in the predictive roles of TILs for pCR rate.

**Table 2:** Sensitivity analysis of TILs and TILs subset.

	Degree of heterogeneity (I2 statistics; %)	P value of heterogeneity	OR (95% CI)	P value of OR
***TILs***				
Total	41.1	0.083	3.93(3.26–4.73)	0.000
**Study design**				
Prospective	23.4	0.250	3.87(3.19–4.69)	0.000
Retrospective	72.3	0.027	4.85(2.42–9.74)	0.000
**Ethnicity**				
Europe	23.4	0.250	3.87(3.19–4.69)	0.000
Asia	72.3	0.027	4.85(2.42–9.74)	0.000
**Sample size**				
≤100			55.91(6.63–471.06)	0.000
>100	15.1	0.308	3.85(3.20–4.64)	0.000
**Study quality**				
Score≤4	86.1	0.007	5.20(1.51–17.94)	0.009
Score>4	12.5	0.330	3.97(3.22–4.88)	0.000
**Cutoff criteria**				
others	62.4	0.021	4.08(3.12–5.33)	0.000
10%	0	0.577	3.80(2.94–4.92)	0.000
**Location**				
Intratumoral sites	0	0.586	4.15(2.95–5.84)	0.000
Stromal sites	0	0.457	3.58(2.50–5.13)	0.000
Both sites	69.7	0.010	4.01(3.03–5.32)	0.000
***CD8***				
Total	0	0.533	6.44(2.52–16.46)	0.000
**Country**				
Japan			5.87(1.35–25.64)	0.019
Korea	18.8	0.267	6.87(2.04–23.15)	0.002
**Cutoff criteria**				
median	0	0.542	8.10(2.83–23.17)	0.000
10%			2.65(0.33–21.10)	0.357
**Location**				
Intratumoral sites	0	0.542	8.10(2.83–23.17)	0.000
Stromal sites			2.65(0.33–21.10)	0.357
***FOXP3-pre NAC***				
Total	69.8	0.019	2.94(1.05–8.26)	0.041
**Country**				
Japan	88.7	0.003	2.30(0.24–21.75)	0.468
Korea	0	0.742	3.90(1.66,9.16)	0.041
**Sample size**				
≤100			0.73(0.26–2.09	0.562
>100	0	0.642	4.95(2.53–9.68)	0.000
**Cutoff criteria**				
median	79.7	0.007	2.84(0.70–11.54)	0.144
10%			3.33(0.93–11.92)	0.065
**Location**				
Intratumoral sites	79.7	0.007	2.84(0.70–11.54)	0.144
Stromal sites			3.33(0.93–11.92)	0.065
***FOXP3-post NAC***				
Total	39.3	0.192	0.41(0.21–0.80)	0.009
**Ethnicity**				
Europe			0.07(0.01–0.53)	0.010
Asia	0	0.838	0.51(0.25–1.03)	0.059
**Sample size**				
≤100	67.8	0.078	0.35(0.14–0.91)	0.030
>100			0.47(0.18–1.22)	0.122
**Cutoff criteria**				
median	0	0.838	0.51(0.25–1.03)	0.059
Score>0			0.07(0.01–0.53)	0.010
**Study quality**				
Score≤4	67.8	0.078	0.35(0.14–0.91)	0.030
Score>4			0.47(0.18–1.22)	0.122

**Figure 8:**
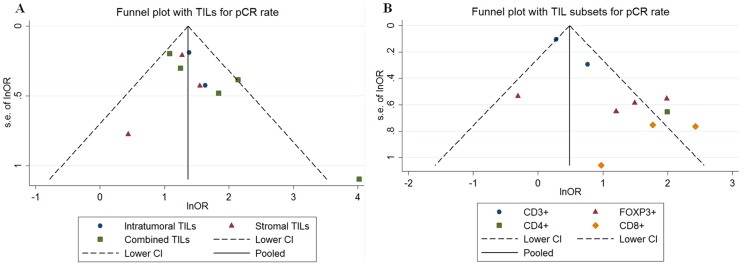
Funnel plot for publication bias in the pooled pCR analysis based on TIL status (A) and TILs subset before (B) treatment.

A funnel plot, Egger's test and Begg's test were performed to assess the publication bias of the selected studies for the pooled pCR rate analysis. The shapes of the funnel plots revealed little evidence of asymmetry for pooled pCR analysis using the TILs level. Egger's test (P = 0.093) and Begger's test (P = 0.152) provided no publication bias in these 8 studies (Fig. 8A). For the comparison of pCR rate in different TILs subset, there was some evidence of asymmetry in the funnel plot before (Fig. 8B) treatment. However, only Egger's test (P = 0.008) before treatment was significant (S4 Table). For TILs in post-NAC breast tissue, there were only three studies, so we did not do the funnel plot, and the Egger's test (P = 0.179) and Begger's test (P = 1.000) provided no publication bias.

## Discussion

This systematic review and meta-analysis suggested that higher level of TILs in pre-NAC biopsy increased about 4 times of pCR rate, whether or not they were detected in stromal, in intra-tumoral or in both sites in univariate analysis. Considering age, tumor size, tumor type, grade, hormonal receptor status, lymph node status or therapeutic regimens, the multivariate analysis indicated that TILs infiltration was still an independent marker for higher pCR rate.

Since immunosuppression has been found in tumor development and progression, immunotherapy has attracted the interest of investigators. Historically, a number of parameters have been assessed as biomarkers for the host immune response in the tumor microenvironment. TILs have been an effective biomarker of anti-tumor immune response in a wide range of cancers and indicated improved overall survival in epithelial ovarian carcinoma [4], [5], colorectal cancer [44], endometrial cancer [6]–[10] and breast cancer [11]–[14]. Compared to adjuvant chemotherapy, neoadjuvant chemotherapy has been proved has been shown to have an equivalent effect on overall survival [16], [45]–[47]. Moreover, pCR, which is the most significant prognostic factor in breast cancer patients with neoadjuvant therapy, is considered to be a valid surrogate marker for overall survival and progression-free survival. More importantly, the neoadjuvant setting provides us with an opportunity to rapidly assess the therapeutic efficacy of a given regimen. Therefore, many studies have analyzed the relationship between the pCR rate and TILs in the neoadjuvant setting.

In an early study, the authors found that the number of intra-tumor CD3+ TILs from pre-treatment biopsy were significantly higher in patients who had pCR after neoadjuvant chemotherapy [48]. Since this study contained a limited sample, the predictive role of TILs in the pathologic response to NAC was subsequently confirmed with a large patient cohort of patients [25]. Depending on the infiltrating sites, TILs may be separated into intratumoral TILs, where lymphocytes have infiltrated in tumor bed (in direct contact with tumors) and stromal TILs, where lymphocytes have infiltrated the tumor stroma [48]. Independent of the training cohort (GeparDuo trial, n = 218), in the validation cohort (GeparTrio trial, n = 840), a higher percentage of intratumoral TILs was an independent maker of a higher pCR rate. They also found that stromal and intratumoral TILs had the predictive value to indicate a better pathological response in the validation cohort. Other studies [31], [42], [43] also found that TILs in stroma still could predict treatment response in a neoadjuvant setting. Five studies [25], [26], [33], [34], [41] reported that higher TILs numbers in both intra-tumoral and stromal sites in pre-treatment biopsy indicated a higher pCR rate. These results were consistent with our meta-analysis that a high number of TILs is a significant predictor of the pCR rate in response to neoadjuvant chemotherapy, whether they were detected in stromal, in intra-tumoral or both sites. For most of the studies we included, the median or more than 10% as a cutoff value, although some studies used increments of 10% [25], [26], [42], [43] or LPBC [25], [26], [41] as a cutoff. We also analyzed these studies, and the results showed that increment 10% of TILs in stomal or intratumorall sites or LPBC patients indicated an increase in the pCR rate in univariate and multivariate analysis. The sensitivity analysis indicated the robustness of the OR estimates. We found no publication bias in the TILs analysis.

As well as we know that, patients with different subtypes of breast cancer have different responses to NAC. A low pCR rate was observed in HR+ and HER2- patients [49]–[51], while a markedly higher pCR rate was reported in HER2+ or triple-negative breast cancer (TNBC) patients [52]–[54]. Whether TILs played different roles in different subtypes of breast cancer remains unknown. We further analyzed the predictive roles of TILs in subtypes of breast cancer. These results indicated that TILs detected in pre-treatment biopsy indicated about 2.5 and 5 times of pCR rate increase in triple negative and HER2 positive breast cancer patients, respectively, but not higher pCR rate in ER positive patients. Previous studies have mostly supported our analysis. Ono et al. [31] found a significant association between pCR and the TILs number in triple-negative patients, but not in other breast cancer subtypes. West et al. [33] also found that higher TILs numbers detected by eight-gene expression were related to the pCR rate in triple negative and HER2 positive patients. Although Yamaguchi et al. [34] found TILs detected by IHC were not a statistically significant correlate to pCR in triple negative patients, only a limited number of patients were included in this study. Our pooled analysis showed that higher levels of lymphocyte infiltration in TNBC and HER2+ patients indicated a better pathologic response to NAC. For multivariate, one study found TILs in ER- [33] and HER2- [26] patients were an independent marker for pCR considering age, tumor size, grade and node status.

TILs subset in breast cancer, primarily CD3+, CD4+, CD8 and (regulatory T-lymphocytes expressing forkhead box P 3 protein) FOXP3+ lymphocytes, have also been studied in the relationship of the pCR rate. Since TILs subset have been shown to change after NAC, we found that higher level of CD8+, CD4+ T and FOXP3+ T lymphocytes in pre-treatment biopsy were correlated with the pCR rate, while CD3+ and lymphocytes in pre-treatment biopsy was not predictive in univariate analysis. Considering age, HR status, HER2 status or Ki67 level, all these subsets were not independent markers for pCR rate. And these finding came from limited studies. Therefore, we have analyzed this result with caution. In post-treatment breast tissue, we found that higher levels of FOXP3+ T lymphocytes infiltration predicted a lower pCR rate in univariate and multivariate analysis. There was none study that showed that the relationship between other T lymphocytes infiltration in post-NAC breast tissue and the pCR rate. The sensitivity analysis indicated sample size, cutoff criteria, study quality or study origin (country) affected the robustness of the OR estimates in TILs subset. Therefore, we should interpret these results with caution and we still need more prospective studies to confirm these results.

In addition to the presence of TILs subset, other ratios or changes were also reported in previous studies. Ladoire et al. [27] found that a reduced Foxp3/CD8 ratio after neoadjuvant chemotherapy was strongly associated with the pCR rate. In their study, CD3+ and CD8+ infiltrates remained stable during the treatment, while FOXP3+ infiltrate strongly declined, suggesting that Treg cells were more sensitive to the chemotherapeutic regimen than were conventional T cells. García-Martínez et al. [55] reported on the relationship between the changes in TILs subset and pCR rate. This study demonstrated that changes greater than the median in CD3+ and CD4+ TILs subset indicated a reduced chance for pCR in response to NAC. These observations suggested that patients with pCR experienced little change in CD3+ and CD4+ T lymphocyte infiltration after NAC, while CD8+ and FOXP3+ T lymphocytes changes were not predictive. Considering limited study reported the relationship between the pCR rate and ratios or changes in TILs subset before and after NAC, more prospective studies were needed in future.

Due to the small number of studies in each subgroup panel, the results of this analysis should be interpreted with caution. Our systematic review has some limitations. First, this is a meta-analysis of published trials, and only six studies included a randomized design, while four studies had a prospective design. The included studies cover a mixed population of operable and locally advanced breast cancer with different prognoses and responses to NAC. Second, different cell scoring methods resulted in bias regarding the assignment of high and low lymphocyte infiltration. In our meta-analysis, cutoff points were used in several ways, with some studies choosing more than 10%, while other studies used the median, quartiles or various scores and related statistics. These differences might be responsible for the variability in reaching a standard threshold for the lymphocyte count. Moreover, in the analysis of TIL, most studies used H&E staining and one study used an 8-gene signature. Third, a limited number of studies showed the correlation between TILs subset and the pCR rate in preciously intratumoral or stromal sites, even in different subtypes of breast cancer. Therefore, there should be more prospective studies to identify these relationships. Moreover, other possible factors, such as hormonal receptor status, grade and ki67 also affect the pCR rate. However, those potentially confounding variables varied considerably among individuals and thus yielded inconsistent prognostic results; multivariate analysis was performed in only a few of the included studies to obtain more precise estimates by adjusting for clinicopathological variables, and we also evaluated these ORs in our meta-analysis. Fourth, some studies concerning TILs subset combined multiple markers and indicated that a particular combination of markers was a more sensitive predictor for recurrence and survival than a single T lymphocyte type, which was identified in intra-tumoural CD8/FOXP3 ratios. However, this finding must be investigated further because of the limited number of studies. Fifth, this is a literature-based analysis, and the majority of included trials are retrospective in nature. Finally, the NAC schemes comprised conventional and nonconventional schedules (e.g., EC-TX epirubicin/cyclophosphamide – docetaxel/capecitabine; EC-T-X epirubicin/cyclophosphamide – docetaxel – capecitabine; PM paclitaxel/non-pegylated liposomal doxorubicin; PMCb paclitaxel/non-pegylated liposomal doxorubicin/carboplatin; and HER2 positive patients with trastuzumab therapy) with slightly different durations. Most studies included both anthracycline- and taxane-based regimens. To the best of our knowledge, the present study represents the largest review ever published concerning TILs and NAC response. These results suggested that a higher TILs level indicated a higher pCR rate for anthracycline- and taxane-based regimens and also platinum- and trastuzumab-containing regimens.

However, the results should be still interpreted with caution, because we may have failed to identify some published and unpublished studies with negative results that would have affected our pooled estimates. Although the funnel plots did not provide evidence of publication bias for pCR stratified by TILs or T lymphocyte subsets, we recognize that the use of relatively few studies may have reduced the power for detecting publication bias.

## Conclusion

In summary, despite the above limitations, our findings suggest that TILs could serve as a robust marker for predicting the pCR rate to NAC, especially in HER2 positive and TNBC patients. In TILs subset, only FOXP3+ lymphocytes had an independent predictive value in post-NAC breast tissue. The predictive role of CD3+ and CD4+ lymphocytes was still unclear due to a limited number of studies addressing these markers. Future studies should use a prospective study design to improve the quality of clinical data and should also consider the clinic pathological variables of the patient, including the tumor grade, ki67, and other tumor microenvironment factors. TILs subset from different locations, breast cancer subtypes and specific neoadjuvant chemotherapy regimens should also be investigated in future studies.

## Supporting Information

S1 Table Risk of bias assessment.
(DOC)

S2 Table Original data from included study (1).
(DOCX)

S3 Table Original data from all included studies (2).
(DOCX)

S4 Table Results of publication bias by Egger's and Begg's tests.
(DOCX)

S1 Checklist PRISMA 2009 checklist.
(DOC)

## References

[1] Siegel R, Ma J, Zou Z, Jemal A (2014) Cancer statistics, 2014. CA Cancer J Clin 64(1):9–29. 24399786 10.3322/caac.21208

[2] Andre F, Dieci MV, Dubsky P, Sotiriou C, Curigliano G, et al (2013) Molecular pathways:involvement of immune pathways in the therapeutic response and outcome in breast cancer. Clin Cancer Res 19(1):28–33. 23258741 10.1158/1078-0432.CCR-11-2701

[3] Caras I, Grigorescu A, Stavaru C, Radu D, Mogos I, et al (2004) Evidence for immune defects in breast and lung cancer patients. Cancer Immunol Immunother 53(12):1146–1152. 15185014 10.1007/s00262-004-0556-2 PMC11034324

[4] Tomsova M, Melichar B, Sedlakova I, Steiner I (2008) Prognostic significance of CD3+ tumor-infiltrating lymphocytes in ovarian carcinoma. Gynecol Oncol 108(2):415–420. 18037158 10.1016/j.ygyno.2007.10.016

[5] Zhang L, Conejo-Garcia JR, Katsaros D, Gimotty PA, Massobrio M, et al (2003) Intratumoral T cells, recurrence, and survival in epithelial ovarian cancer. N Engl J Med 348(3):203–213. 12529460 10.1056/NEJMoa020177

[6] De Jong RA, Leffers N, Boezen HM, van der Zee AGJ, Hollema H, et al (2009) Presence of tumor-infiltrating lymphocytes is an independent prognostic factor in type I and II endometrial cancer. Gynecol Oncol 114(1):105–110. 19411095 10.1016/j.ygyno.2009.03.022

[7] Giatromanolaki A, Bates GJ, Koukourakis MI, Sivridis E, Gatter KC, et al (2008) The presence of tumor-infiltrating FOXP3+ lymphocytes correlates with intratumoral angiogenesis in endometrial cancer. Gynecol Oncol 110(2):216–221. 18533240 10.1016/j.ygyno.2008.04.021

[8] Ino K, Yamamoto E, Shibata K, Kajiyama H, Yoshida N, et al (2008) Inverse correlation between tumoral indoleamine 2,3-dioxygenase expression and tumor-infiltrating lymphocytes in endometrial cancer: its association with disease progression and survival. Clin Cancer Res 14(8):2310–2317. 18413819 10.1158/1078-0432.CCR-07-4144

[9] Kondratiev S, Sabo E, Yakirevich E, Lavie O, Resnick MB (2004) Intratumoral CD8+ T lymphocytes as a prognostic factor of survival in endometrial carcinoma. Clin Cancer Res 10(13):4450–4456. 15240536 10.1158/1078-0432.CCR-0732-3

[10] Yamagami W, Susumu N, Tanaka H, Hirasawa A, Banno K, et al (2011) Immunofluorescence-detected infiltration of CD4+ FOXP3+ regulatory T cells is relevant to the prognosis of patients with endometrial cancer. Int J Gynecol Cancer 21(9):1628–1634. 21897268 10.1097/IGC.0b013e31822c271f

[11] Loi S, Sirtaine N, Piette F, Sagado R, Viale G, et al (2013) Prognostic and predictive value of tumor-infiltrating lymphocytes in a phase III randomized adjuvant breast cancer trial in node-positive breast cancer comparing the addition of docetaxel to doxorubicin with doxorubicin-based chemotherapy: BIG 02-98. J Clin Oncol 31(7):860–867. 23341518 10.1200/JCO.2011.41.0902

[12] Mahmoud SM, Paish EC, Powe DG, Macmillan RD, Grainge MJ, et al (2011) Tumor-infiltrating CD8+ lymphocytes predict clinical outcome in breast cancer. J Clin Oncol 29(15):1949–1955. 21483002 10.1200/JCO.2010.30.5037

[13] Menard S, Tomasic G, Casalini P, Balsari A, Pilotti S, et al (1997) Lymphoid infiltration as a prognostic variable for early-onset breast carcinomas. Clin Cancer Res 3(5):817–819. 9815754

[14] Mohammed ZM, Going JJ, Edwards J, Elsberger B, Doughty JC, et al (2012) The relationship between components of tumour inflammatory cell infiltrate and clinicopathological factors and survival in patients with primary operable invasive ductal breast cancer. Br J Cancer 107(5):864–873. 22878371 10.1038/bjc.2012.347 PMC3426752

[15] Bear HD, Anderson S, Brown A, Smith R, Mamounas EP, et al (2003) The effect on tumor response of adding sequential preoperative docetaxel to preoperative doxorubicin and cyclophosphamide: preliminary results from National Surgical Adjuvant Breast and Bowel Project Protocol B-27. J Clin Oncol 21(22):4165–4174. 14559892 10.1200/JCO.2003.12.005

[16] Fisher B, Brown A, Mamounas E, Wieand S, Robidoux A, et al (1997) Effect of preoperative chemotherapy on local-regional disease in women with operable breast cancer: findings from National Surgical Adjuvant Breast and Bowel Project B-18. J Clin Oncol 15(7):2483–2493. 9215816 10.1200/JCO.1997.15.7.2483

[17] Ismael G, Hegg R, Muehlbauer S, Heinzmann D, Lum B, et al (2012) Subcutaneous versus intravenous administration of (neo)adjuvant trastuzumab in patients with HER2-positive, clinical stage I-III breast cancer (HannaH study): a phase 3, open-label, multicentre, randomised trial. Lancet oncol 13(9):869–878. 22884505 10.1016/S1470-2045(12)70329-7

[18] Fisher CS, Ma CX, Gillanders WE, Aft RL, Eberlein TJ, et al (2012) Neoadjuvant chemotherapy is associated with improved survival compared with adjuvant chemotherapy in patients with triple-negative breast cancer only after complete pathologic response. Ann Surg Oncol 19(1):253–258. 21725686 10.1245/s10434-011-1877-y PMC3892697

[19] Kong X, Moran MS, Zhang N, Haffty B, Yang Q (2011) Meta-analysis confirms achieving pathological complete response after neoadjuvant chemotherapy predicts favourable prognosis for breast cancer patients. Eur J Cancer 47(14):2084–2090. 21737257 10.1016/j.ejca.2011.06.014

[20] Liedtke C, Mazouni C, Hess KR, André F, Tordai A, et al (2008) Response to neoadjuvant therapy and long-term survival in patients with triple-negative breast cancer. J Clin Oncol 26(8):1275–1281. 18250347 10.1200/JCO.2007.14.4147

[21] Denkert C, Loibl S, Muller BM, Eidtmann H, Schmitt WD, et al (2013) Ki67 levels as predictive and prognostic parameters in pretherapeutic breast cancer core biopsies: a translational investigation in the neoadjuvant GeparTrio trial. Ann oncol 24(11):2786–2793. 23970015 10.1093/annonc/mdt350

[22] Fasching PA, Heusinger K, Haeberle L, Niklos M, Hein A, et al (2011) Ki67, chemotherapy response, and prognosis in breast cancer patients receiving neoadjuvant treatment. BMC cancer 11:486.. 22081974 10.1186/1471-2407-11-486 PMC3262864

[23] Ohno S, Chow LW, Sato N, Masuda N, Sasano H, et al (2013) Randomized trial of preoperative docetaxel with or without capecitabine after 4 cycles of 5-fluorouracil- epirubicin-cyclophosphamide (FEC) in early-stage breast cancer: exploratory analyses identify Ki67 as a predictive biomarker for response to neoadjuvant chemotherapy. Breast Cancer Res Treat 142(1):69–80. 24122389 10.1007/s10549-013-2691-y PMC3825616

[24] Aruga T, Suzuki E, Saji S, Horiguchi S, Horiguchi K, et al (2009) A low number of tumor-infiltrating FOXP3-positive cells during primary systemic chemotherapy correlates with favorable anti-tumor response in patients with breast cancer. Oncol Rep 22(2):273–278. 19578766

[25] Denkert C, Loibl S, Noske A, Roller M, Müller BM, et al (2010) Tumor-associated lymphocytes as an independent predictor of response to neoadjuvant chemotherapy in breast cancer. J Clin Oncol 28(1):105–113. 19917869 10.1200/JCO.2009.23.7370

[26] Issa-Nummer Y, Darb-Esfahani S, Loibl S, Kunz G, Nekljudova V, et al (2013) Prospective validation of immunological infiltrate for prediction of response to neoadjuvant chemotherapy in HER2-negative breast cancer–a substudy of the neoadjuvant GeparQuinto trial. PloS One 8(12):e79775.. 24312450 10.1371/journal.pone.0079775 PMC3846472

[27] Ladoire S, Arnould L, Apetoh L, Coudert B, Martin F, et al (2008) Pathologic complete response to neoadjuvant chemotherapy of breast carcinoma is associated with the disappearance of tumor-infiltrating foxp3+ regulatory T cells. Clin Cancer Res 14(8):2413–2420. 18413832 10.1158/1078-0432.CCR-07-4491

[28] Liu F, Li Y, Ren M, Zhang X, Guo X, et al (2012) Peritumoral FOXP3(+) regulatory T cell is sensitive to chemotherapy while intratumoral FOXP3(+) regulatory T cell is prognostic predictor of breast cancer patients. Breast Cancer Res Treat 135(2):459–467. 22842982 10.1007/s10549-012-2132-3

[29] Loi S, Sirtaine N, Piette F, Salgado R, Viale G, et al (2013) Prognostic and predictive value of tumor-infiltrating lymphocytes in a phase III randomized adjuvant breast cancer trial in node-positive breast cancer comparing the addition of docetaxel to doxorubicin with doxorubicin-based chemotherapy: BIG 02-98. J Clin Oncol 31(7):860–867. 23341518 10.1200/JCO.2011.41.0902

[30] Oda N, Shimazu K, Naoi Y, Morimoto K, Shimomura A, et al (2012) Intratumoral regulatory T cells as an independent predictive factor for pathological complete response to neoadjuvant paclitaxel followed by 5-FU/epirubicin/cyclophosphamide in breast cancer patients. Breast Cancer Res Treat136(1):107–116.. 10.1007/s10549-012-2245-8 22986814

[31] Ono M, Tsuda H, Shimizu C, Yamamoto S, Shibata T, et al (2012) Tumor-infiltrating lymphocytes are correlated with response to neoadjuvant chemotherapy in triple-negative breast cancer. Breast Cancer Res Treat 132(3):793–805. 21562709 10.1007/s10549-011-1554-7

[32] Seo AN, Lee HJ, Kim EJ, Kim HJ, Jang MH, et al (2013) Tumour-infiltrating CD8+ lymphocytes as an independent predictive factor for pathological complete response to primary systemic therapy in breast cancer. Br J Cancer 09(10):2705–2713. 10.1038/bjc.2013.634 PMC3833219 24129232

[33] West NR, Milne K, Truong PT, Macpherson N, Nelson BH, et al (2011) Tumor-infiltrating lymphocytes predict response to anthracycline-based chemotherapy in estrogen receptor-negative breast cancer. Breast Cancer Res13(6):R126.. 10.1186/bcr3072 PMC3326568 22151962

[34] Yamaguchi R, Tanaka M, Yano A, Tse GM, Yamaguchi M, et al (2012) Tumor-infiltrating lymphocytes are important pathologic predictors for neoadjuvant chemotherapy in patients with breast cancer. Human Pathol 43(10):1688–1694. 22516244 10.1016/j.humpath.2011.12.013

[35] Loi S, Michiels S, Salgado R, Sirtaine N, Jose V, et al (2014) Tumor infiltrating lymphocytes are prognostic in triple negative breast cancer and predictive for trastuzumab benefit in early breast cancer: results from the FinHER trial. Ann Oncol 25(8):1544–1550. 24608200 10.1093/annonc/mdu112

[36] Mahmoud SM, Paish EC, Powe DG, Macmillan RD, Grainge MJ, et al (2011) Tumor-infiltrating CD8+ lymphocytes predict clinical outcome in breast cancer. J Clin Oncol 29(15):1949–1955. 21483002 10.1200/JCO.2010.30.5037

[37] Mcshane LM, Altman DG, Sauerbrei W, Taube SE, Gion M, et al (2005) REporting recommendations for tumour MARKer prognostic studies (REMARK). Br J Cancer 93(4):387–391. 16106245 10.1038/sj.bjc.6602678 PMC2361579

[38] Hayes DF, Ethier S, Lippman ME (2006) New guidelines for reporting of tumor marker studies in breast cancer research and treatment: REMARK. Breast Cancer ResTreat 100(2):237–238. 10.1007/s10549-006-9253-5 16773436

[39] Egger M, Davey Smith G, Schneider M, Minder C (1997) Bias in meta-analysis detected by a simple graphical test. BMJ 315:629–634. 9310563 10.1136/bmj.315.7109.629 PMC2127453

[40] Begg CB, Mazumdar M (1994) Operating characteristics of a rank correlation test for publication bias. Biometrics 50:1088–1010. 7786990

[41] Denkert C, Loibl S, Salat C, Sinn BV, Schem C, et al**.** (2013) Increased tumor-associated lymphocytes predict benefit froom additioin of carboplatin to neoadjuvant therapy for triple-negative and HER2-positive early breast cancer in the GeparSixto trial (GBG66/AGO-B). Cancer Res 73 (24 Suppl): Abstract nr S1-06.

[42] Lee HJ, Seo JY, Ahn JH, Ahn SH, Gong G (2013) Tumor-associated lymphocytes predict response to neoadjuvant chemotherapy in breast cancer patients. J Breast Cancer 16(1):32–39. 23593079 10.4048/jbc.2013.16.1.32 PMC3625767

[43] Loi S, Jose VL, Bono P, Sirtaine N, Jose V, et al. (2013) Tumor infiltrating lymphocytes (TIL) indecate trastuzumab benefit in early-stage HER2-positive breast cancer. Cancer Res 73 (24 Suppl): Abstract nr S1-05.

[44] Mei Z, Liu Y, Liu C, Cui A, Liang Z, et al (2014) Tumour-infiltrating inflammation and prognosis in colorectal cancer: systematic review and meta-analysis. Br J Cancer 110(6):1595–1605. 24504370 10.1038/bjc.2014.46 PMC3960618

[45] Fisher B, Bryant J, Wolmark N, Mamounas E, Brown A, et al (1998) Effect of preoperative chemotherapy on the outcome of women with operable breast cancer. J Clin Oncol 16(8):2672–2685. 9704717 10.1200/JCO.1998.16.8.2672

[46] Mauri D, Pavlidis N, Ioannidis JP (2005) Neoadjuvant versus adjuvant systemic treatment in breast cancer: a meta-analysis. J Natl Cancer Inst 97(3):188–194. 15687361 10.1093/jnci/dji021

[47] Van Der Hage JA, Van De Velde CJ, Julien JP, Tubiana-Hulin M, Vandervelden C, et al (2001) Preoperative chemotherapy in operable breast cancer: results from the European Organization for Research and Treatment of Cancer trial 10902. J Clin Oncol 19(22):4224–4237. 11709566 10.1200/JCO.2001.19.22.4224

[48] Hornychova H, Melichar B, Tomsova M, Mergancova J, Urminska H, et al (2008) Tumor-infiltrating lymphocytes predict response to neoadjuvant chemotherapy in patients with breast carcinoma. Cancer Inves 26(10):1024–1031. 10.1080/07357900802098165 19093260

[49] Bhargava R, Beriwal S, Dabbs DJ, Ozbek U, Soran A, et al (2010) Immunohistochemical surrogate markers of breast cancer molecular classes predicts response to neoadjuvant chemotherapy: a single institutional experience with 359 cases. Cancer 116(6):1431–1439. 20131351 10.1002/cncr.24876

[50] Straver ME, Rutgers EJ, Rodenhuis S, Linn SC, Loo CE, et al (2010) The relevance of breast cancer subtypes in the outcome of neoadjuvant chemotherapy. Ann Sur Oncol 17(9):2411–2418. 10.1245/s10434-010-1008-1 PMC2924493 20373039

[51] Tan MC, Al Mushawah F, Gao F, Aft RL, Gillanders WE, et al (2009) Predictors of complete pathological response after neoadjuvant systemic therapy for breast cancer. Am J Surg 198(4):520–525. 19800460 10.1016/j.amjsurg.2009.06.004 PMC3924770

[52] Carey LA, Dees EC, Sawyer L, Gatti L, Moore DT, et al (2007) The triple negative paradox: primary tumor chemosensitivity of breast cancer subtypes. Clin Cancer Res 13(8):2329–2334. 17438091 10.1158/1078-0432.CCR-06-1109

[53] Melichar B, Hornychova H, Kalabova H, Bašová H, Mergancová J, et al (2012) Increased efficacy of a dose-dense regimen of neoadjuvant chemotherapy in breast carcinoma: a retrospective analysis. Med oncol 29(4):2577–2585. 22392196 10.1007/s12032-012-0195-y

[54] Montagna E, Bagnardi V, Rotmensz N, Viale G, Pruneri G, et al (2010) Pathological complete response after preoperative systemic therapy and outcome: relevance of clinical and biologic baseline features. Breast Cancer Res Treat 124(3):689–699. 20625816 10.1007/s10549-010-1027-4

[55] García-Martínez E, Luengo-Gil G, Chaves A, Gonzalez-Billalabeitia E, García GT, et al**.** (2013) Changes induced by neoadjuvant chemotherapy (NCT) in breast cancer infiltrating lymphocytes (TIL) subpopulations are associated with chemo-sensitivity and prognosis. Cancer Res 73 (24 Suppl): P5-01-08.

