# Correction: The Value of Tumor Infiltrating Lymphocytes (TILs) for Predicting Response to Neoadjuvant Chemotherapy in Breast Cancer: A Systematic Review and Meta-Analysis

**DOI:** 10.1371/journal.pone.0119243

**Published:** 2015-03-03

**Authors:** The PLOS ONE Staff

In Fig. 4, there is an extra symbol next to “stroma,” and the P values for “Both Sites” are incorrect. Please view the correct Fig. 4 here.

**Fig 4:**
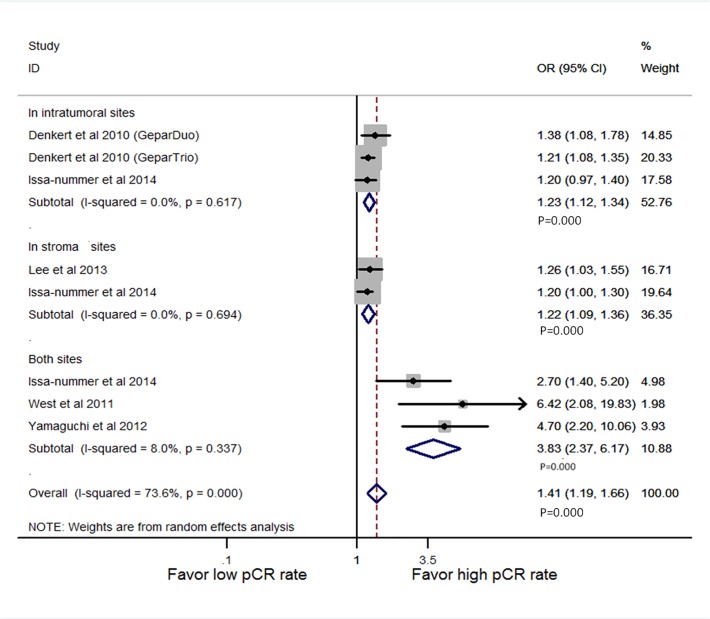
Forest plots from the random-effect meta-analysis of the efficacy of TILs on the NAC response stratified by infiltration locations in multivariate way. The width of horizontal line represents 95% CI of the individual studies, and the grey boxes represent the weight of each study. The diamond represents the overall summary estimate. The unbroken vertical line was set at the null value (HR = 1.0).

In Fig. 6, the P value is incorrect. Please view the correct Fig. 6 here.

**Fig 6:**
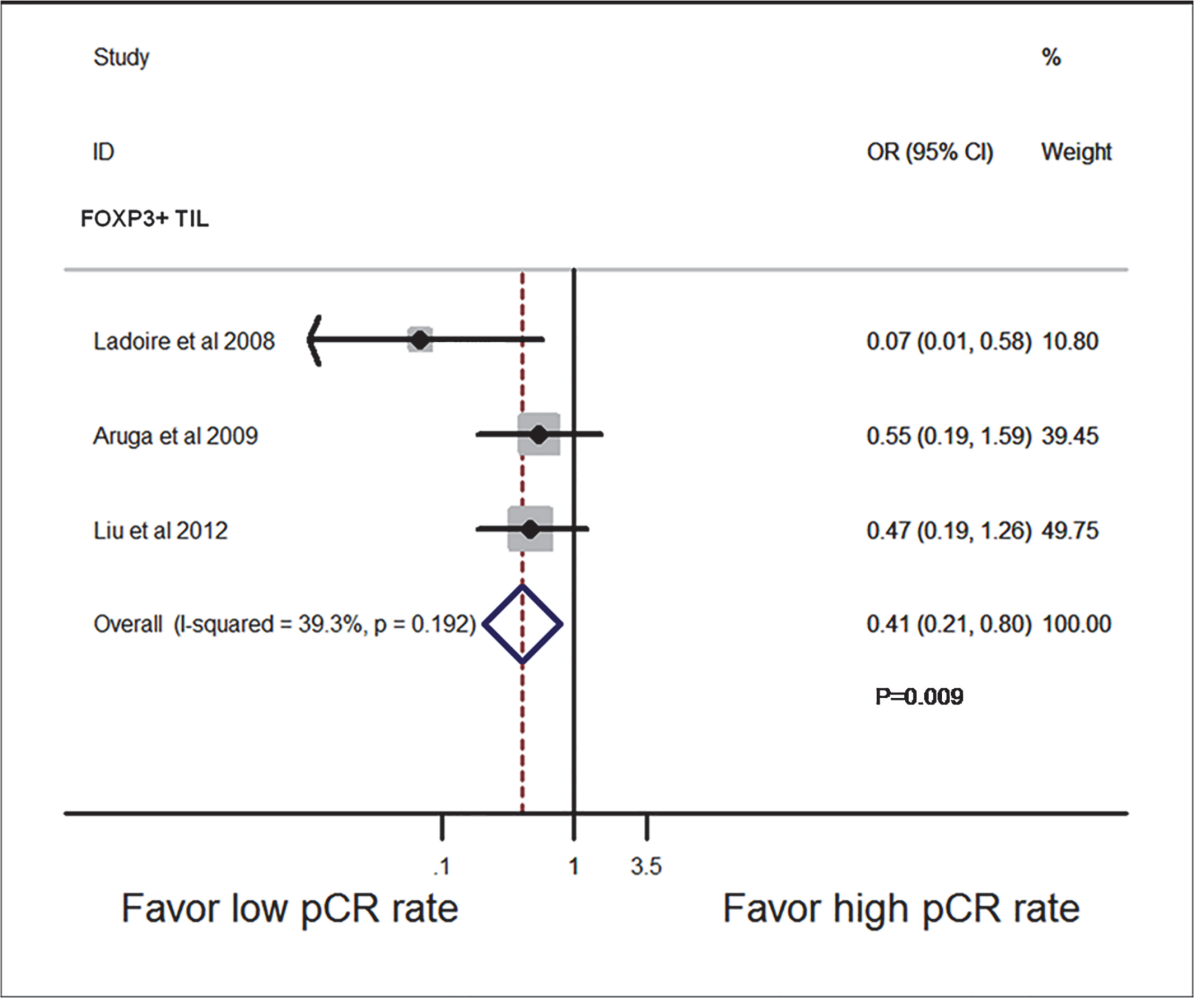
Forest plots from the fixed-effect meta-analysis of the efficacy of TILs subset on NAC response in post-treatment breast tissue. The width of horizontal line represents 95% CI of the individual studies, and the grey boxes represent the weight of each study. The diamond represents the overall summary estimate. The unbroken vertical line was set at the null value (HR = 1.0).
