# Supplementary material for: The Value of Tumor Infiltrating Lymphocytes (TILs) for Predicting Response to Neoadjuvant Chemotherapy in Breast Cancer: A Systematic Review and Meta-Analysis
Source: PLoS One. 2014 Dec 12;9(12):e115103. doi: 10.1371/journal.pone.0115103 (PMC4264870; doi:10.1371/journal.pone.0115103)
Supplement: S1 Table — Risk of bias assessment. (DOC) [file pone.0115103.s001.doc]

**Table S1. Risk of Bias Assessment**

| **Study** | **Is the population under study defined with in- and exclusion criteria?** | **Were patient data prospectively collected?** | **Are the main prognostic patient and tumor characteristics presented?^1^** | **Is the method used for determination of protein expression specified?** | **Is the IHC or HE staining protocol specified?^2^** | **Were stainings evaluated by > 1 observer?** | **Is the study endpoint defined?** | **Is the time of follow up specified?** | **Is loss during analysis or follow up described?** | **Quality rating** |
| --- | --- | --- | --- | --- | --- | --- | --- | --- | --- | --- |
| Ladoire et al 2008 [27] | 0 | 0 | 1 | 1 | 1 | 1 | 0 |  | 0 | 4 |
| Aruga et al 2009 [24] | 0 | 0 | 0 | 1 | 1 | 0 | 1 |  | 1 | 4 |
| Denkert et al 2010 [25] GeparDuo | 1 | 1 | 1 | 0 | 1 | 1 | 1 |  | 1 | 7 |
| Denkert et al 2010 [25] GeparTrio | 1 | 1 | 1 | 0 | 1 | 0 | 1 |  | 1 | 6 |
| West et al 2011 [33] | 1 | 1 | 1 | 0 | 0 | 0 | 1 |  | 1 | 5 |
| Oda et al 2012 [30] | 0 | 0 | 1 | 1 | 1 | 0 | 1 |  | 0 | 4 |
| Ono et al 2012 [31] | 0 | 0 | 1 | 0 | 1 | 1 | 1 |  | 1 | 5 |
| Yamaguchi et al 2012 [34] | 0 | 0 | 1 | 0 | 1 | 1 | 0 |  | 0 | 3 |
| Liu et al 2012 [28] | 0 | 0 | 1 | 1 | 1 | 1 | 1 |  | 0 | 5 |
| Seo et al 2013 [32] | 0 | 0 | 1 | 1 | 1 | 0 | 1 |  | 0 | 4 |
| Lee et al 2013 [42] | 0 | 0 | 1 | 1 | 1 | 0 | 1 |  | 0 | 4 |
| Loi et al 2013 [43] | 1 | 1 | 1 | 0 | 1 | 0 | 1 |  | 0 | 5 |
| Denkert et al 2013 [41] | 1 | 1 | 1 | 0 | 1 | 1 | 1 |  | 0 | 6 |
| Issa-Nummer et al 2014 [26] | 1 | 1 | 1 | 0 | 1 | 0 | 1 |  | 0 | 5 |

Abbreviations: IHC = immunohistochemistry; HE staining=Hematoxylin-eosin staining^1^At least four of the following characteristics: age at diagnosis, TNM stage, tumor type, differentiation grade and residual tumor after primary surgery. ^2^At least four of the following criteria: antigen retrieval, primary antibody, dilution, detection method, cut-off value for positive expression.
