# Supplementary material for: The Value of Tumor Infiltrating Lymphocytes (TILs) for Predicting Response to Neoadjuvant Chemotherapy in Breast Cancer: A Systematic Review and Meta-Analysis
Source: PLoS One. 2014 Dec 12;9(12):e115103. doi: 10.1371/journal.pone.0115103 (PMC4264870; doi:10.1371/journal.pone.0115103)
Supplement: S2 Table — Original data from included study (1). (DOCX) [file pone.0115103.s002.docx]

**Table S2. Original data from included study (1)**

| Study | Time | Marker | pCR high | pCR low | non-pCR high | non-pCR low | Subtype | OR(95% CI) |
| --- | --- | --- | --- | --- | --- | --- | --- | --- |
| Ladoire 2008[27] | **post-NAC** | **FOXP3** | **1** | **11** | **25** | **19** | **Total** | **0.07(0.01-0.58)** |
| Aruga 2009 [24] | **pre-NAC** | **FOXP3** | **8** | **10** | **36** | **33** | **Total** | **0.73(0.26-2.08)** |
|  | **post-NAC** | **FXOP3** | **7** | **11** | **37** | **32** | **Total** | **0.55(0.19-1.59)** |
| Denkert 2010(GeparTrio)[25] | **pre-NAC** | **CD3** | **48** | **52** | **30** | **70** | **Total** | **2.15(1.21-3.85)** |
|  |  | **iTILs** | **79** | **64** | **165** | **532** | **Total** | **3.98(2.74-5.78)** |
|  |  | **sTILs** | **108** | **35** | **323** | **374** | **Total** | **3.57(2.37-5.38)** |
| Denkert 2010 (GeparDuo)[25] | **pre-NAC** | **iTILs** | **14** | **14** | **31** | **159** | **Total** | **5.13(2.23-11.82)** |
| Ono 2012 [31] | **pre-NAC** | **TILs** |  |  |  |  |  |  |
|  |  |  | **7** | **2** | **16** | **17** | **HR-/HER2+** | **3.72(0.67-20.63)** |
|  |  |  | **1** | **2** | **7** | **36** | **HR+/HER2-** | **2.57(0.20-32.39)** |
|  |  |  | **33** | **8** | **65** | **74** | **Total** | **4.70(2.02-10.89)** |
| Yamaguchi 2012[34] | **pre-NAC** | **TILs** | **4** | **0** | **3** | **9** | **TNBC** | **20（0.93-429.90）** |
|  |  |  | **7** | **0** | **3** | **4** | **HR-/HER2+** | **12.29（0.80-466.24）** |
|  |  |  | **4** | **1** | **1** | **5** | **HR+/HER2+** | **24.43（1.03-580.63）** |
| Denkert 2013 [41] | **pre-NAC** | **TILs(LPBC)** | **85** | **147** | **57** | **291** | **Total** | **2.95(2.00-4.36)** |
|  |  |  | **51** | **90** | **38** | **135** | **TNBC** | **2.01(1.22-3.31)** |
|  |  |  | **34** | **58** | **19** | **155** | **HER2+** | **4.78(2.53-9.05)** |
| Lee 2013 [42] | **pre-NAC** | **TILs** | **17** | **2** | **132** | **24** | **Total** | **1.55(0.34-7.13)** |
|  |  | **CD8+** | **18** | **1** | **136** | **20** | **Total** | **2.65(0.33-20.93)** |
|  |  | **FOXP3** | **16** | **3** | **96** | **60** | **Total** | **3.33(0.93-11.92)** |
