# Supplementary material for: The Value of Tumor Infiltrating Lymphocytes (TILs) for Predicting Response to Neoadjuvant Chemotherapy in Breast Cancer: A Systematic Review and Meta-Analysis
Source: PLoS One. 2014 Dec 12;9(12):e115103. doi: 10.1371/journal.pone.0115103 (PMC4264870; doi:10.1371/journal.pone.0115103)
Supplement: S3 Table — Original data from all included studies (2). (DOCX) [file pone.0115103.s003.docx]

**Table S3. Original data from all included studies (2)**

| Study | Marker | Ratio | Cutoff value | Uni- or Multi variate analysis | Time | OR（95% CI） | | | Data source | Population |
| --- | --- | --- | --- | --- | --- | --- | --- | --- | --- | --- |
|  |  |  |  |  |  | **Intratumor** | **Stroma** | **Both sites** |  |  |
| Ladoire 2008[27] | **FXOP3** | **H vs L** | **Score>0** | **univariate** | **post-NAC** | **0.07(0.01-0.58)** |  |  | **original data** | **Total** |
| Aruga 2009 [24] | **FXOP3** | **H vs L** | **Median** | **univariate** | **pre-NAC** | **0.73(0.26-2.08)** |  |  | **original data** | **Total** |
|  | **FXOP3** | **H vs L** | **Median** | **univariate** | **post-NAC** | **0.550(0.19-1.59)** |  |  | **original data** | **Total** |
| Denkert 2010 (GeparDuo)[25] | **TILs** | **H vs L** | **10%** | **univariate** | **Pre-NAC** | **5.13(2.23-11.82)** |  |  | **original data** | **Total** |
|  | **TILs** | **H vs L** | **Per 10% increase** | **univariate** | **pre-NAC** | **1.54(1.28-1.86)** |  |  | **paper direct** | **Total** |
|  | **TILs** | **H vs L** | **Per 10% increase** | **multivariate** | **pre-NAC** | **1.38(1.08-1.78)** |  |  | **paper direct** | **Total** |
|  | **TILs** | **H vs L** | **Per 10% increase** | **univariate** | **pre-NAC** |  | **1.37(1.17-1.60)** |  | **paper direct** | **Total** |
| Denkert 2010 (GerparTrio)[25] | **TILs** | **H vs L** | **Per 10% increase** | **univariate** | **pre-NAC** | **1.36(1.26-1.47)** |  |  | **paper direct** | **Total** |
|  | **TILs** | **H vs L** | **Per 10% increase** | **multivariate** | **pre-NAC** | **1.21(1.08-1.35)** |  |  | **paper direct** | **Total** |
|  | **TILs** | **H vs L** | **Per 10% increase** | **univariate** | **pre-NAC** |  | **1.27(1.19-1.35)** |  | **paper direct** | **Total** |
|  | **TILs** | **H vs L** | **10%** | **univariate** | **pre-NAC** | **3.98(2.74-5.78)** |  |  | **original data** | **Total** |
|  | **TILs** | **H vs L** | **10%** | **univariate** | **pre-NAC** |  | **3.57(2.37-5.38)** |  | **original data** | **Total** |
|  | **TILs** | **H vs L** | **focal infiltration** | **univariate** | **pre-NAC** |  |  | **2.339(1.18-4.62)** | **paper direct** | **Total** |
|  | **TILs** | **H vs L** | **LPBC** | **univariate** | **pre-NAC** |  |  | **8.53(4-18.2)** | **paper direct** | **Total** |
|  | **CD3+** | **H vs L** | **10%** | **univariate** | **pre-NAC** | **2.15(1.21-3.85)** |  |  | **original data** | **Total** |
| West 2011[33] | **TILs** | **H vs L** | **upper quarTILs e** | **univariate** | **pre-NAC** |  |  | **6.33(2.49-16.08)** | **paper direct** | **ER-** |
|  | **TILs** | **H vs L** | **upper quarTILs e** | **multivariate** | **pre-NAC** |  |  | **6.42(2.08-19.83)** | **paper direct** | **ER-** |
|  | **TILs** | **H vs L** | **upper quarTILs e** | **univariate** | **pre-NAC** |  |  | **11.25(1.145-110.5)** | **paper direct** | **HER2+** |
|  | **TILs** | **H vs L** | **upper quarTILs e** | **univariate** | **pre-NAC** |  |  | **6.333(2.124-18.89)** | **paper direct** | **TNBC** |
| Ono 2012 [31] | **TILs** | **H vs L** | **score>2** | **univariate** | **pre-NAC** |  | **3.12(0.96-10.15)** |  | **paper direct** | **TNBC** |
|  | **TILs** | **H vs L** | **score>2** | **multivariate** | **pre-NAC** |  | **2.78(0.84-9.18)** |  | **paper direct** | **TNBC** |
|  | **TILs** | **H vs L** | **score>2** | **univariate** | **pre-NAC** |  | **3.72(0.67-20.63)** |  | **original data** | **HR-/HER2+** |
|  | **TILs** | **H vs L** | **score>2** | **univariate** | **pre-NAC** |  | **2.57(0.20-32.39)** |  | **original data** | **HR+/HER2-** |
|  | **TILs** | **H vs L** | **score>2** | **univariate** | **pre-NAC** |  | **4.70(2.02-10.89)** |  | **original data** | **Total** |
| Yamaguchi 2012 [34] | **TILs** | **H vs L** | **score>1** | **univariate** | **pre-NAC** |  |  | **55.9(6.63-470.8)** | **paper direct** | **Total** |
|  | **TILs** | **H vs L** | **score>1** | **multivariate** | **pre-NAC** |  |  | **4.7(2.2-10.06)** | **paper direct** | **Total** |
|  | **TILs** | **H vs L** | **score>1** | **univariate** | **pre-NAC** |  |  | **20（0.93-429.90）** | **original data** | **TNBC** |
|  | **TILs** | **H vs L** | **score>1** | **univariate** | **pre-NAC** |  |  | **12.29（0.80-466.24）** | **original data** | **HR-/HER2+** |
|  | **TILs** | **H vs L** | **score>1** | **univariate** | **pre-NAC** |  |  | **24.43（1.03-580.63）** | **original data** | **HR+/HER2+** |
| Oda 2012 [30] | **CD8+** | **H vs L** | **meidan** | **univariate** | **pre-NAC** | **5.873(1.345-25.641)** |  |  | **paper direct** | **Total** |
|  | **CD8+** | **H vs L** | **meidan** | **multivariate** | **pre-NAC** | **1.368(0.236-7.941)** |  |  | **paper direct** | **Total** |
|  | **FXOP3** | **H vs L** | **meidan** | **univariate** | **pre-NAC** | **7.268(2.453-21.533)** |  |  | **paper direct** | **Total** |
|  | **FXOP3** | **H vs L** | **meidan** | **multivariate** | **pre-NAC** | **6.251(1.448-26.994)** |  |  | **paper direct** | **Total** |
| Liu 2012 [28] | **FXOP3** | **H vs L** | **meidan** | **univariate** | **post-NAC** | **2.11(0.19-1.26)** |  |  | **paper direct** | **Total** |
|  | **FXOP3** | **L vs H** | **meidan** | **univariate** | **post-NAC** |  | **3.90(0.09-0.75)** |  | **paper direct** | **Total** |
|  | **FXOP3** | **H vs L** | **meidan** | **multivariate** | **post-NAC** |  | **2.80(0.13-0.95)** |  | **paper direct** | **Total** |
| Seo 2013 [32] | **CD4** | **H vs L** | **meidan** | **univariate** | **pre-NAC** | **7.328(2.019-26.206)** |  |  | **paper direct** | **Total** |
|  | **CD4** | **H vs L** | **meidan** | **multivariate** | **pre-NAC** | **2.377(0.483-11.688)** |  |  | **paper direct** | **Total** |
|  | **CD8+** | **H vs L** | **meidan** | **univariate** | **pre-NAC** | **11.288(2.518-50.610)** |  |  | **paper direct** | **Total** |
|  | **CD8+** | **H vs L** | **meidan** | **multivariate** | **pre-NAC** | **9.786(2.121-45.149)** |  |  | **paper direct** | **Total** |
|  | **FOXP3** | **H vs L** | **meidan** | **univariate** | **pre-NAC** | **4.444(1.411-13.999)** |  |  | **paper direct** | **Total** |
|  | **FOXP3** | **H vs L** | **meidan** | **multivariate** | **pre-NAC** | **0.292(0.039-2.212)** |  |  | **paper direct** | **Total** |
| Lee 2013 [42] | **TILs** | **H vs L** | **Per 10% increase** | **univariate** | **pre-NAC** |  | **1.32（1.08-1.60）** |  | **paper direct** | **Total** |
|  | **TILs** | **H vs L** | **Per 10% increase** | **multivariate** | **pre-NAC** |  | **1.26（1.03-1.55）** |  | **paper direct** | **Total** |
|  | **TILs** | **H vs L** | **Per 10% increase** | **univariate** | **pre-NAC** |  | **1.08（0.84-1.38）** |  | **paper direct** | **HER2+** |
|  | **TILs** | **H vs L** | **10%** | **univariate** | **pre-NAC** |  | **1.55(0.34-7.13)** |  | **original data** | **Total** |
|  | **CD3** | **H vs L** | **Per 10% increase** | **univariate** | **pre-NAC** |  | **1.32(1.08-1.62)** |  | **paper direct** | **Total** |
|  | **CD8+** | **H vs L** | **Per 10% increase** | **univariate** | **pre-NAC** |  | **1.29(1.06-1.58)** |  | **paper direct** | **Total** |
|  | **CD8+** | **H vs L** | **10%** | **univariate** | **pre-NAC** |  | **2.65(0.33-20.93)** |  | **original data** | **Total** |
|  | **FOXP3** | **H vs L** | **counts** | **univariate** | **pre-NAC** |  | **1.02(1.01-1.04)** |  | **paper direct** | **Total** |
|  | **FOXP3** | **H vs L** | **20%** | **univariate** | **pre-NAC** |  | **3.33(0.93-11.92)** |  | **original data** | **Total** |
| Loi 2013 [43] | **TILs** | **H vs L** | **Per 10% increase** | **univariate** | **pre-NAC** |  | **1.16（1.01-1.32）** |  | **paper direct** | **HER2+** |
| Denkert 2013 [41] | **TILs** | **H vs L** | **LPBC** | **univariate** | **pre-NAC** |  |  | **2.95(2.00-4.36)** | **original data** | **ER-** |
|  | **TILs** | **H vs L** | **LPBC** | **univariate** | **pre-NAC** |  |  | **2.01(1.22-3.31)** | **original data** | **TNBC** |
|  | **TILs** | **H vs L** | **LPBC** | **univariate** | **pre-NAC** |  |  | **4.78(2.53-9.05)** | **original data** | **HER2+** |
| Issa-Nummer 2014 [26] | **TILs** | **H vs L** | **Per 10% increase** | **univariate** | **pre-NAC** | **1.2(1.1-1.5)** |  |  | **paper direct** | **HER2-** |
|  | **TILs** | **H vs L** | **Per 10% increase** | **multivariate** | **pre-NAC** | **1.2(0.97-1.4)** |  |  | **paper direct** | **HER2-** |
|  | **TILs** | **H vs L** | **Per 10% increase** | **univariate** | **pre-NAC** |  | **1.2(1.1-1.4)** |  | **paper direct** | **HER2-** |
|  | **TILs** | **H vs L** | **Per 10% increase** | **multivariate** | **pre-NAC** |  | **1.2(1.0-1.3)** |  | **paper direct** | **HER2-** |
|  | **TILs** | **H vs L** | **LPBC** | **univariate** | **pre-NAC** |  |  | **3.5(1.9-6.2)** | **paper direct** | **HER2-** |
|  | **TILs** | **H vs L** | **LPBC** | **multivariate** | **pre-NAC** |  |  | **2.7(1.4-5.2)** | **paper direct** | **HER2-** |
