# Supplementary material for: The Value of Tumor Infiltrating Lymphocytes (TILs) for Predicting Response to Neoadjuvant Chemotherapy in Breast Cancer: A Systematic Review and Meta-Analysis
Source: PLoS One. 2014 Dec 12;9(12):e115103. doi: 10.1371/journal.pone.0115103 (PMC4264870; doi:10.1371/journal.pone.0115103)
Supplement: S4 Table — Results of publication bias by Egger's and Begg's tests. (DOCX) [file pone.0115103.s004.docx]

**Table S4. Egger’s and Begg’s tests of all studies**

**Egger’s test Begg’s test**

**Intercept p value Kendall’s τ p value**

**TILs 1.91 0.093 17 0.152**

**Pre-treatment 3.5 0.008 13 0.283**

**Post-treatment -3.46 0.179 -1 1.000**
